# A comprehensive risk assessment method for Christmas tree combined with a multi source information fusion algorithm and an improved HAZOP method

**DOI:** 10.1371/journal.pone.0339897

**Published:** 2026-01-05

**Authors:** Qiong Wang, Huimin Li, Guanlong Ren

**Affiliations:** 1 School of Architecture and Engineering, Zhanjiang University of Science and Technology, Zhanjiang, Guangdong, China; 2 School of Education Management, Assumption University, Bangkok, Thailand; 3 School of Management, Xi’an University of Architecture and Technology, Xi’an, Shaanxi, China; 4 CNOOC Zhanjiang Branch Company, Zhanjiang, Guandong, China; University of Bonab, IRAN, ISLAMIC REPUBLIC OF

## Abstract

The Christmas tree faces significant safety risks during installation and requires extremely high reliability during operation. In this study, a comprehensive risk assessment method combined GA-BP neural network and improved HAZOP method is established, which is the first application used in the field of risk analysis for offshore oil equipment. The sample risk data during the installation and operation of the China’s first domestically produced Christmas tree is extracted through multi-source fusion, and the possibility and severity levels of risk occurrence are classified and judged. By training and testing the model it shows that the average error rate of the proposed GA-BP multi-source information fusion method is only 5.10%. Through risk data extraction and evaluation, the failure probability when the Christmas tree lowering into water is found to be the highest, with a critical importance coefficient of 0.24. No high-risk failure modes are found during the production process, but there are 36 moderate risk points. Deep water gas well field testing shows the theoretical judgment is consistent with the actual discovery, and with using of hydrate inhibitor injection the blockage risk at the nozzle of the Christmas tree is alleviated, which verifying the accuracy of the method. The method has improved the risk warning system for the safe operation of the Christmas tree, and can provide technical support for similar subsea oil and gas production equipment.

## 1. Introduction

Deep water areas contain abundant oil and gas resources, and the extraction of offshore oil and gas requires various sub-sea production equipment. However, the special natural environment and complex oil and gas storage conditions bring many technical challenges and safety operation risks during the development of deep water oil and gas fields [1,2]. The sub-sea production system centered on sub-sea Christmas tree is an indispensable key equipment in the sub-sea development mode of offshore deep water oil and gas fields. It can connect facilities such as sub-sea wellheads and manifolds, control the flow of oil and gas from the well, and monitor oil and gas well parameters such as production pressure, annulus pressure, temperature, sand production and water content, etc. During the normal production period of deep water oil and gas wells, the Christmas tree is subjected to complex working conditions such as high temperature, high pressure, and high erosion, as well as communication processes such as hydraulic and electrical systems. At the same time, the sub-sea Christmas tree is subject to ocean currents and corrosion in the marine environment, and may be affected by underwater earthquakes, which the risk of external failure is increased significantly [3].

During the normal operation of the Christmas tree, various types of sensors need to be installed on the tree to accurately and timely obtain the operating status and production parameters [4]. The status monitoring data of the Christmas tree is transmitted to the upper control system through the subsea control module (SCM). The parameters from different positions, times, forms and characteristics of the Christmas tree is diverse, complex and difficult to process, and also with a large amount of uncertainty. At the same time, various parameters in different production processes are collected, correlated, feature processed, state evaluated, and various types of information interact and fuse with each other, which brings difficulties to accurate identification and control of the information, thereby posing risks to the safe operation of the Christmas tree [5]. Therefore, the normal operation process of the Christmas tree can be regarded as a typical multi-source information fusion processing and decision-making process. By recognizing, synthesizing and judging various data, the fusion of multi-source information can achieve the processing of various received parameters, and then comprehensively analyze the data based on experience or relevant theoretical knowledge to make the final judgment.

Multi-source information fusion refers to the integration, analysis and processing of data and information from multiple different sources to improve the accuracy and efficiency of decision-making. Based on this, combined with risk assessment methods, the accurately obtained data analysis results are classified into risk likelihood and severity levels, so as to evaluate the safety risks of oil and gas equipment such as the Christmas tree in the production process, and comprehensively obtain safety risk assessment results, and ensure the normal operation of the Christmas tree and the safe production of oil and gas wells [6]. At present, multi-source information fusion technology has been widely applied in various fields such as fault diagnosis, intelligent transportation, weather forecasting, image processing, and positioning. It is a multi-faceted and multi-level processing process, including the detection, combination, estimation, and correlation of multi-source data, in order to improve the accuracy of state estimation and complete system evaluation timely [7]. This technology can fully utilize multiple information sources, combine redundant and complementary information in time and space according to certain optimization criteria, and provide consistent descriptions or interpretations of the observation environment to generate new fusion results. Its goal is to derive more effective information by optimizing the combination of various separated parameters, and to improve the performance of the entire system. The methods used for multi-source information fusion mainly include estimation theory methods, uncertainty reasoning methods, and artificial intelligence methods. The genetic algorithm (GA) used in this article is a random search and optimization method based on the principles of biological evolution. It simulates the genetic and evolutionary processes in nature, generates new individuals through operations such as survival of the fittest, crossover, and mutation in the population, and continuously iterates to find the optimal solution [8]. Thakkar et al. [9] used genetic algorithms to optimize and select different features in stock price and trend prediction, which improves the correlation of features and reducing redundant information.. Kande et al. [10] proposed an optimized framework based on genetic algorithm, and combined system dynamics modeling, machine learning, and swarm intelligence optimization algorithms to achieve intelligent scheduling and management of heterogeneous multi asset sets. Liang et al. [11] proposed an optimal data fusion method based on genetic algorithm, which effectively addresses the probability fusion problem of highly conflicting given sources. Guo et al. [12] proposed a genetic algorithm based optimization mode for multi-source traffic information collection and combination, which is more accurate in achieving the optimization process of multi-source traffic information collection and combination, and has a better degree of information fusion. Sun et al. [13] proposed an improved genetic algorithm for multi-sensor data fusion, which significantly improves the accuracy and stability of data fusion and has a shorter execution time.

Neural networks are computer programs designed to simulate the information processing methods in the human nervous system. They can fuse data captured by multiple sensors, recognize patterns and relationships through learning, and use them for tasks such as classification, regression, and clustering to achieve more accurate and comprehensive environmental perception and prediction [14,15]. Guo et al. [16] proposed an statistically modified convolutional neural network (SMCNN) to improve the accuracy of GNSS-R wind speed detection. By fusing the reflected signals of different polarization directions and satellite signals, and adopting a statistical correction strategy, the accuracy and robustness of wind speed detection are further improved. Passos et al. [17] proposed a multimodal audio and video information fusion method based on canonical correlation graph neural networks. It utilizes audio and video data captured by multiple sensors and fuses them together using graph neural networks for speech enhancement, which can significantly reduce the consumption of computing resources and energy and improve the enhancement effect. Gao et al. [18] proposed an information fusion method based on adaptive convolutional neural networks for facial expression recognition. Different feature information is obtained in the fully connected layer for information fusion, which has higher recognition accuracy in facial expression recognition.

At present, the petroleum exploration and development field is accelerating the development of energy digitization and intelligence, which integrate technologies such as big data, cognitive computing, and artificial intelligence, and achieving efficiency improvement and cost reduction through digital and intelligent transformation. Shi et al. [19] designed a multi-source information fusion function model for oil monitoring and analyzed a multi-source information fusion algorithm suitable for oil monitoring information. Gong et al. [20] constructed an ontology based multi-source information fusion framework in the petroleum field, and proposed an ontology based element similarity algorithm and fusion rules based on this framework. Through experimental analysis, it can improve the efficiency of multi-source petroleum data analysis. Gao [21] correlated, organized and fused multi-source data in the geological research of Daqing Oilfield, established structural models, sedimentary facies models, reservoir models and realized the application of multi-source oilfield data fusion methods in oil and gas geological research and decision-making. Ma [22] studied the information sources and expression methods in petroleum exploration and development, analyzed their relationship with drilling geological characteristic parameters and constructed a multidimensional heterogeneous spatial model for petroleum exploration and development. Yin et al. [23] developed an early monitoring method for underground gas invasion based on non volume expansion principle and an early monitoring equipment for gas invasion based on multi-source information fusion, which solved the problem of delayed gas invasion disposal and high risk of blowout caused by traditional well killing methods that require drilling stoppage and well closure. Qiang et al. [24] established a genetic algorithm optimized BP neural network model for predicting horizontal well productivity in ultra-low permeability sandstone reservoirs. The prediction error of various parameters is reduced by 75% compared to the standard BP model. Guo et al. [25] used an adaptive GA-BP neural network to predict the node pressure in the oilfield water injection pipeline network system. The simulation results showed that its prediction accuracy improved by 11.7% compared to the traditional GA-BP model. Li [26] established a GA-BP rotary drilling rig drilling efficiency prediction model, and calculated the model accuracy through two sets of test data during the construction process. The overall model accuracy reached over 80%.

In terms of safety risk assessment of oil and gas equipment, Xiao et al. [27] used AHP method to construct an evaluation index system for the Christmas tree sea trials, and applied fuzzy analysis method to evaluate its comprehensive performance. Ping et al. [28] used the layer of protection analysis (LOPA) method to semi quantitatively analyze the safety integrity level (SIL) classification of typical Christmas tree processes, ensuring the safe and efficient operation of sub-sea production systems. Zhu et al. [29] used the fuzzy fault tree analysis method to conduct reliability analysis on the Christmas tree system, and further studied the failure mechanism. They established a Markov model for the Christmas tree system and quantitatively analyzed its reliability. Det Norske Veritashas developed a series of assessment software for offshore oil engineering, mainly including Neptune Offshore® for risk assessment and hazard assessment in offshore oil and gas development equipment, Leak® for calculating the frequency of various petrochemical plant accidents, and SAFETI® for conducting multifunctional risk assessment and hazard assessment [30]. However, there have been no reports on the application of multi-source information fusion methods in the field of sub-sea oil and gas equipment production, and there has been no relevant research on the safety operation risks of sub-sea oil and gas production equipment combined with artificial intelligence methods.

Given the particularity of subsea oil and gas production process, a multi-source information fusion state estimation method based on artificial intelligence is introduced, and the genetic algorithm is used to optimize the initial weights and thresholds of the BP neural network. Then a GA-BP neural network fusion design is carried out and a GA-BP neural network fusion algorithm is constructed. Based on the Christmas tree monitoring system model with established GA-BP multi-source information fusion method and the accurate identification of the Christmas tree operation risk data, an improved HAZOP method is established by combining the risk matrix analysis method to evaluate the risks of different nodes during the installation and production process of the Christmas tree. Finally a comprehensive risk assessment method for the safe operation of subsea oil and gas equipment is proposed, which can significantly improve the prediction ability of the multi-source information during the subsea oil and gas production process.

## 2. Description of the Christmas tree system

### 2.1. Christmas tree

**Fig 1:**
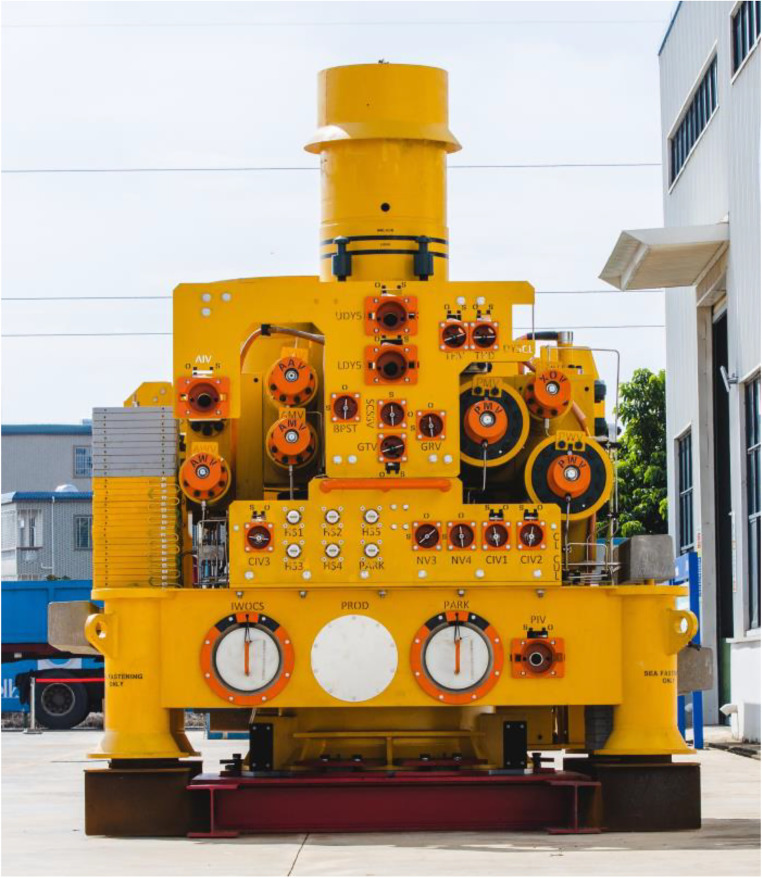
A horizontal Christmas tree.

The Christmas tree is the core component of sub-sea oil and gas production equipment, and is also the foundation for building subsea oil and gas production system. Fig 1 shows a perspective view of the China’s first domestically produced Christmas tree. It mainly consists of the tree body, tubing hanger, valves, pipelines and wellhead connectors. The oil and gas flow through the wellhead, tubing hanger, production channel, throttle valve, and then transport to the subsea production manifold.

Various parameters and status monitored during the operation of the Christmas tree are achieved through sensors. The specific monitoring functions implemented include:

(1) Monitor the annular pressure
(2) Monitor production pressure to adjust production flow rate
(3) Monitor the opening of production control valves (if any)
(4) Monitor the temperature upstream and downstream of the production control valve
(5) Monitor the sand content of oil
(6) Monitor sealing performance
(7) Monitor the temperature and pressure downhole

The parameters during the normal production of the Christmas tree mainly include temperature, pressure, and sand concentration; temperature and pressure downhole; the working status signal of the flow control module, the flow rate and pressure of the SCM, the dual coil solenoid valve signal controlled by subsea electronic modules(SEM), the pilot liquid pressure that maintains the working position of the solenoid valve, the chemical injection metering valve signal controlled by SEM, the production main valve signal controlled by SEM, the pressure and flow rate related to the valve actuator, etc. The specific monitoring points of the Christmas tree are shown in Table 1.

**Table 1:** Monitoring points on the Christmas tree.

No.	Sensor	Description
1	CIMV MEG ECM	Ethylene glycol metering valve electronic control module
2	CIMV MEG OPT	Outlet pressure of ethylene glycol metering valve
3	CIMV MEG IPT	Inlet pressure of ethylene glycol metering valve
4	CIMV MeOH ECM	Methanol metering valve electronic control module
5	CIMV MeOH OPT	Outlet pressure of methanol metering valve
6	CIMV MeOH IPT	Inlet pressure of methanol metering valve
7	CIMV CI OPT	Outlet pressure of corrosion inhibitor metering valve
8	CIMV CI DFPT	Downstream filter pressure of corrosion inhibitor metering valve
9	CIMV CI IPT	Inlet pressure of corrosion inhibitor metering valve
10	WGFM 1	Wet gas flow rate 1
11	ASD	Sound sand detector
12	PI1 A	Production pressure 1 A
13	TI1 A	Production temperature 1 A
14	APT A	Annulus pressure A
15	TI1 B	Production temperature 1 B
16	PI1 B	Production temperature 1 B
17	TI2 B	Production temperature 2 B
18	APT B	Annulus pressure B

The status data of the Christmas tree is transmitted to the upper control system through the SCM. All control signals and data acquisition need to go through SCM, and all sub-sea instruments and sensors need to be connected to SCM. Therefore, as a key component of sub-sea control systems, SCM is responsible for collecting, processing, and transmitting data from all sensors and instruments. The monitoring points related to SCM are shown in Table 2.

**Table 2:** Monitoring points in the SCM.

No.	Sensor	Measured variables	Installation position
1	HPHPT	High head pressure	High pressure pipe head
2	ICVRPT	ICV reflux pressure	ICV reflux pipeline
3	LPHPT	Low head pressure	Low pressure pipe head
4	LP-FM	Low pressure flow rate	Low pressure oil supply pipeline
5	LPR-FM	Low pressure return oil flow rate	Low pressure return oil pipeline
6	HP-FM	High pressure flow rate	High pressure oil supply pipeline
7	HRP-FM	High pressure return oil flow rate	High pressure return oil pipeline

### 2.2. Installation conditions

The installation process of the Christmas tree is carried out in an extremely complex environment, which is not only affected by ocean currents and waves, but also by the movement of the upper platform. Due to the varying environmental loads and boundary conditions during different installation stages, the installation process can be divided into the following stages:

Stage 1: Lift the Christmas tree to the moon pool area of the drilling platform;

Stage 2: Lower through the moon pool area and ultimately traverse the splash zone. This is a critical stage as it involves crossing the sea level. The sea conditions at sea level are relatively harsh compared to those below, which can easily cause sudden lateral forces on the drilling pipe and the Christmas tree. Therefore, the lowering speed should be as slow as possible during this stage;

Stage 3: The main process is to lower the Christmas tree from 50m below sea level to about 50 meters above the wellhead. During this stage, the environmental loads on the Christmas tree and the lowering tools are mainly ocean currents. In this stage, the force forms on the drilling pipe and Christmas tree are relatively uniform, and there are rarely sudden changes. Therefore, the lowering speed is higher compared to Stage 2;

Stage 4: The main process is lowering the last 50m and the placement of the Christmas tree on the wellhead. Due to its proximity to the seabed, the installation in this stage will be affected by the maximum lateral displacement of the drilling pipe, and usually requires the assistance of a remotely operated vehicle(ROV). The movement of the upper platform cannot be ignored, and it may be necessary to adjust the position of the drilling platform to complete the docking between the Christmas tree and the wellhead.

### 2.3. Operation conditions

During the normal production of the Christmas tree, due to the higher temperature of the reservoir oil and gas than the seabed environment, the heat is continuously dissipated to the main body of the Christmas tree under the action of radial temperature difference. Under the pressure of the formation, the main body of the Christmas tree is subjected to load changes caused by temperature and pressure. At the same time, the production channel structure of the Christmas tree is complex, and as the temperature and pressure changes, natural gas hydrates are easily formed at the internal throttle valve, which further increases the load on the Christmas tree. At the same time, there are multiple electro-hydraulic crossing channels installed on the Christmas tree. During the production process, chemical agents such as methanol and ethylene glycol need to be injected through the crossing channels to perform pressure tests on the upper and lower plugs. These operations will have a certain impact on the deformation and contact of the sealing interface of the Christmas tree. In addition, the Christmas tree contains many valve channels inside, and the production fluid may generate local turbulence inside the tree, which may cause fatigue damage to the tree. The tubing hanger inside the Christmas tree needs to consider the stress effects of temperature and pressure changes and tubing weight. At the same time, the wellhead connector connected to the Christmas tree will also generate axial separation force and mechanical load due to internal pressure. The Christmas tree is also subject to ocean currents and corrosion in the marine environment during service which increases the risk of external failure [5,31].

## 3. Method establishment

In order to comprehensively train multiple monitoring parameters during the safe operation of the Christmas tree, and accurately predict abnormal data in the production process, and identify risk factors, the paper optimizes the initial weights and thresholds of the BP neural network using GA to accelerate network convergence, improves the stability of prediction results, and obtains global optimal solutions. Then a multi-source fusion algorithm based on GA optimization of BP neural network is proposed.

### 3.1. Model of GA-BP neural network fusion

#### 3.1.1. Multi-source information fusion of the Christmas tree.

**Fig 2:**
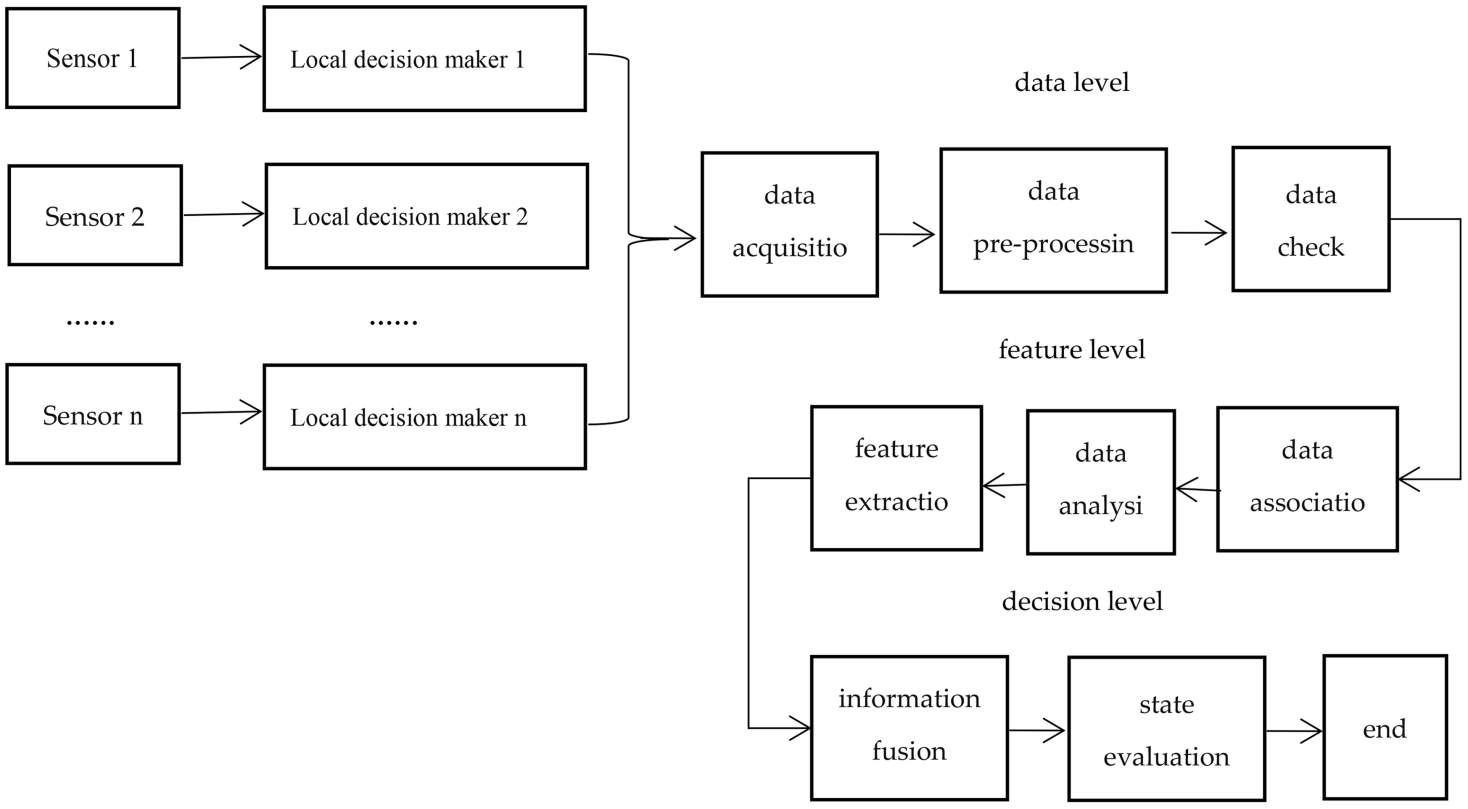
Overall structure of multi-source information fusion.

The fusion process of multi-source information monitored by sensors at the data level, feature level and decision level, from low to high, is as follows: Firstly, the multi-sensor group of the Christmas tree performs pre-processing on the collected raw data, mainly converting non electric parameters collected from multiple sensors or information sources into electric parameters, and then converting the electric information into digital parameters through A/D converters that can be processed by computers. Then, the fusion structure pre-processes the digital parameters, mainly by calibrating, associating and predicting the data, removes interference parameters during the data collection process, and extracts useful information; Thirdly, classify, aggregate, and process digital parameters based on its different features, known as feature extraction; Finally, the extracted feature parameters is sent to the fusion center for information fusion to be comprehensively analyzed and evaluated. The overall structure of multi-source information fusion is shown in Fig 2.

#### 3.1.2. BP neural network design method.

**Fig 3:**
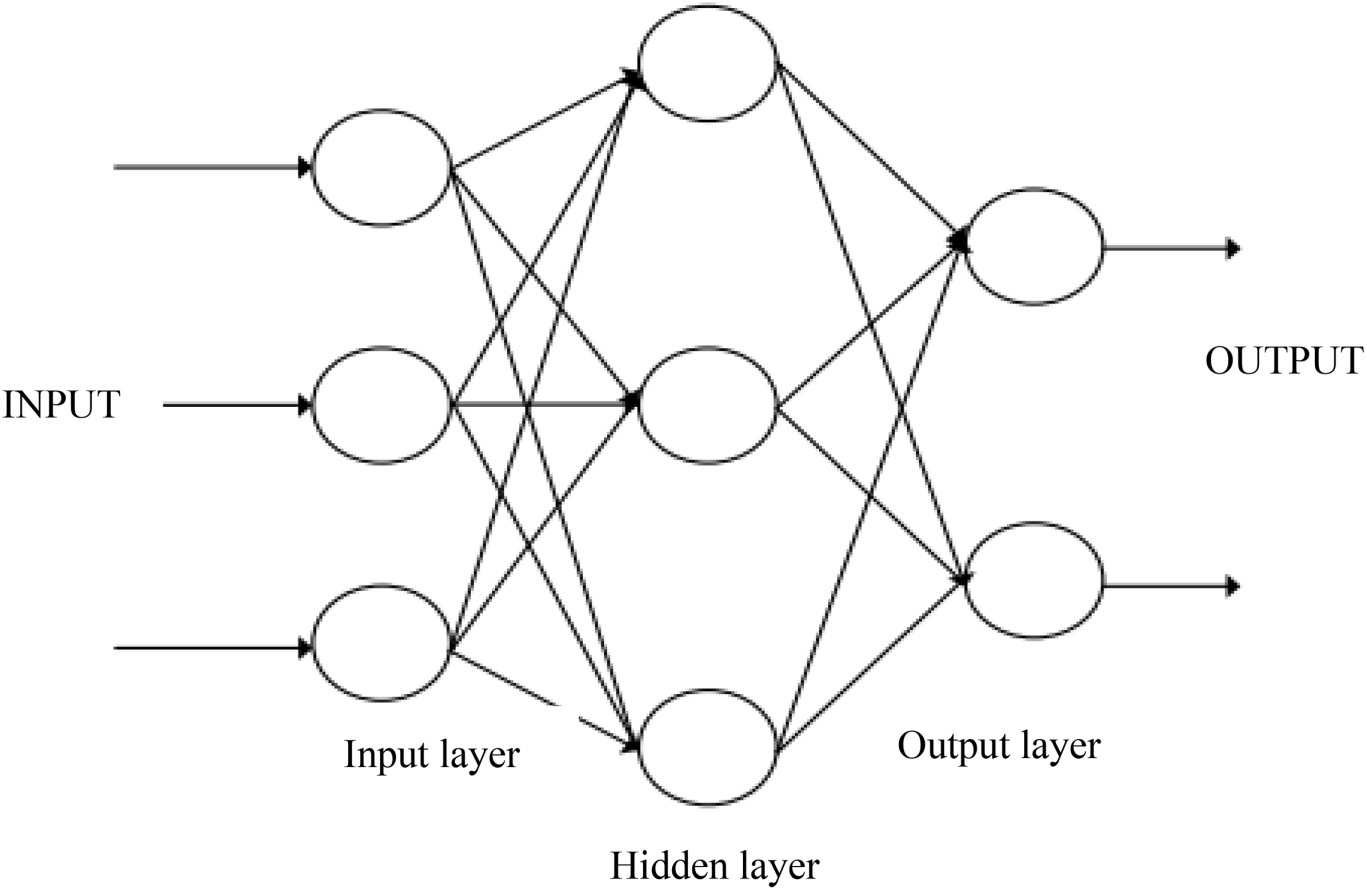
Three layer BP neural network structure.


The typical three-layer BP neural network structure diagram is shown in Fig 3. Assuming that the activation functions of each node in the network are S-shaped functions [15], and the *i-th* node in the input layer is denoted as *net*_i_, the output is denoted as *o*_i_, and the output of the *k-th* node in the output layer is *y*_sk_, then the input of the *j-th* node in the intermediate layer is:

$$\begin{array}{c} {net}_{\text{i}}=\sum {\mathrm{\backslash nolimits}}_{j}w_{\text{ji}}o_{\text{i}} \end{array}$$ (1)

$$o_{\text{i}}=f({net}_{\text{j}})\;$$ (2)

$$\begin{array}{c} {net}_{\text{k}}=\sum {\mathrm{\backslash nolimits}}_{j}w_{\text{kj}}o_{\text{j}} \end{array}$$ (3)

$$y_{\text{sk}}=o_{\text{k}}=f({net}_{\text{k}})$$ (4)

If the error of a network is defined as the difference between the expected output and the actual output, then there is ${\;e}_{\text{k}}=y_{\text{qk}}-y_{\text{sk}}$. If the output layer has *i* neurons, the scoring error between the actual output and the expected output is defined as:


$$\begin{array}{c} E=\frac{\text{1}}{\text{2}}\sum {\mathrm{\backslash nolimits}}_{n=\text{1}}^{i}{(y_{\text{qk}}-y_{\text{sk}})}^{\text{2}} \end{array}$$ (5)

(1) Determination of training sample

The target input of the BP neural network is a five-dimensional matrix composed of monitoring data collected from 9 SCM monitoring points, and the target output is the actual abnormal monitoring data output [32]. The final training sample is shown in Equation (6).


$$\begin{array}{c} \left\{ \begin{array}{c} \;\;\;\;InputData\;=\left[\begin{array}{c} u_{\text{pi}}\\ v_{\text{pi}}\\ x_{\text{pi}}\\ y_{\text{pi}}\\ z_{\text{pi}} \end{array}\right]\\ \;\;\;\;TargetData\;=\left[\begin{array}{c} u_{\text{ri}}\\ v_{\text{ri}}\\ x_{\text{ri}}\\ y_{\text{ri}}\\ z_{\text{ri}} \end{array}\right] \end{array}\right.\;\;\;\;\;\;\;(i=\text{1},\text{2}......n) \end{array}$$ (6)

Where InputData is the input sample, (*u*_pi_, *v*_pi_, *x*_pi_, *y*_pi_, *z*_pi_) is the five-dimensional matrix composed of monitoring data at the *i-th* time point, TargetData is the target output sample, and (*u*_ri_, *v*_ri_, *x*_ri_, *y*_ri_, *z*_ri_) is the real abnormal monitoring data at the *i-th* time point.

(2) Determination of neuron parameters

The empirical formula commonly used to determine the number of hidden layer neurons is shown in Equation (7) [22].


$$m=\sqrt{n+l}+a$$ (7)

Where *m* is the number of hidden layer nodes; *n* is the number of input layer nodes; *l* is the number of output layer nodes; *a* is a constant between 1–10.

#### 3.1.3. GA-BP neural network fusion design.

GA is an optimized method that focuses on populations rather than individuals, which can seek a parallel solution approach. It can search for the optimal solution to a problem from a global perspective, and rely on its powerful global search ability to optimize the initial weights and thresholds of BP neural networks. Therefore, it is very suitable for analyzing and optimizing the large data streams received by the SCM of the Christmas tree. The specific implementation steps are as follows [32]:

(1) Determination of encoding method and initial population

Randomly initialize weights and thresholds. Assuming that the number of input neurons in the neural network is *n*, the number of output layer neurons is *k*, the number of hidden layer neurons is *m*, and the encoding length is *L*, then:


L=n×m+m×k+m+k (8)

(2) Selection of fitness function

The fitness function is generally determined based on the difference between the actual output and the expected output of the network. In this paper, the fitness function is selected as shown in Equation (9).


$$E=\sum_{i=\text{1}}^{n}{(T_{\text{i}}-Y_{\text{i}})}^{\text{2}}$$ (9)

Where *T*_i_, *Y*_i_ respectively represent the actual output and expected output of the *i-th* training sample, and *n* represents the number of training samples.

(3) Determination of selection operators

When optimizing the BP algorithm with genetic algorithm, the roulette wheel method is usually chosen. If the *i-th* individual fitness is *f*_i_, the probability *P*_si_ of the *i-th* individual being left behind is:


$$P_{\text{si}}=\frac{f_{\text{i}}}{\sum_{j=\text{1}}^{n}f_{\text{i}}}$$ (10)

(4) Selection of crossover operators

This article chooses the overall crossover method, as shown in Equation (11).


$$\alpha \;=\left\{ \;\;\;\;\begin{array}{c} \frac{f_{\text{max}}-f_{\text{i}}}{f_{\text{max}}-f_{\text{avg}}}\;f_{\text{i}}\geq f_{\text{avg}}\\ \;\text{0}.\text{35 }f_{\text{i}}<f_{\text{avg}} \end{array}\right.\;$$ (11)

Where  α is the combination coefficient of gene coding, $f_{\text{i}}$ is the fitness value of the *i-th* individual in the parent population, *f*_max_, *f*_avg_ respectively represent the maximum and average fitness values of individuals in the parent population.

(5) Selection of mutation operators

The combination of mutation operator and selection operator can effectively avoid the problem of immature convergence in genetic algorithm [33]. In this paper, the BP neural network mutation model optimized by genetic algorithm is as follows:


$$\omega^{,}=\omega +\beta (\rho -\text{0}.\text{5})E_{\text{max}}$$ (12)

**Fig 4:**
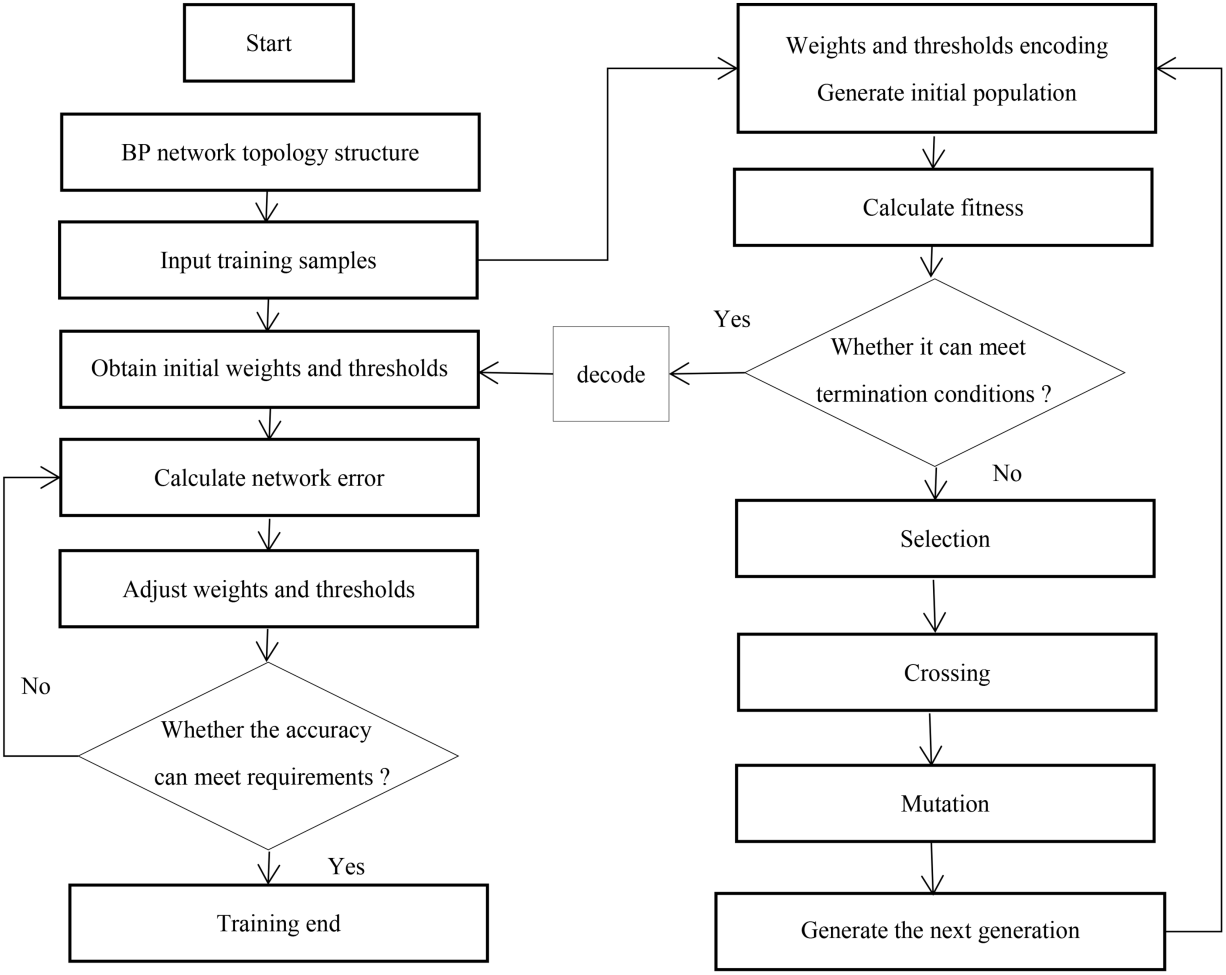
Flow chart of GA-BP neural network.

Where β is the mutation factor; $\omega^{,}$、ω are the network weights and thresholds before and after mutation, ρ is a random number between [0-1], and $E_{\text{max}}$ is the mean square error of the individual with the highest fitness in the parent population, usually taken as values between [0.5–1] [34]. The final GA-BP neural network fusion flowchart is shown in Fig 4.

### 3.2. Method of improved HAZOP

Hazard and Operability Analysis (HAZOP) is a structured analysis method used to identify design defects, process hazards and operational issues. Its focus is on various specific values of the process or operational steps. Its basic process is guided by guiding words to determine the changes (deviations) in the process state, identify the hazards in the equipment and process. In this process, an analysis team composed of various specialties systematically studies each unit (i.e., analysis node) in a prescribed manner, analyzes the hazards and operability issues caused by deviations from the design process conditions [35,36]. The in-service process of the Christmas tree involves complex technological processes, therefore it is advisable to choose the HAZOP method for risk assessment.

However, only using qualitative methods cannot intuitively reflect the risk value, and cannot quantitatively evaluate a single influencing factor which may result in incomplete risk assessment results. A risk matrix is a semi quantitative method with grading the likelihood and generating a certain risk level. It can be applied to analyze potential risks in a project or associated with adopting a certain approach [37,38]. When using the risk matrix for risk assessment, the severity of the consequences is qualitatively classified into several levels, and the likelihood of the risk event occurring is also qualitatively classified into several levels. Then, the severity is showed at the row and the likelihood is showed at the column, and the qualitative risk level is given at the intersection of the rows and columns.

Therefore, based on the accurate prediction of abnormal monitoring data using the GA-BP multi-source information fusion method, the HAZOP method combined with the risk matrix method is used to classify the risk level. The probability and severity of risk occurrence are classified and the risk matrix is used as the basis for risk assessment to evaluate the internal production risks of the Christmas tree. The general form of the improved HAZOP+risk matrix analysis table is shown in Table 3.

**Table 3:** Improved HAZOP+Risk matrix analysis table.

Node Description	Node No.							
Guideword/ Deviation	Possible Causes	Consequences	Safeguards	Probability	Severity	Risk Priority	Recommendations	Remarks

## 4. Case study and discussion

### 4.1. Process construction of multi-source information fusion based on GA-BP

**Fig 5:**
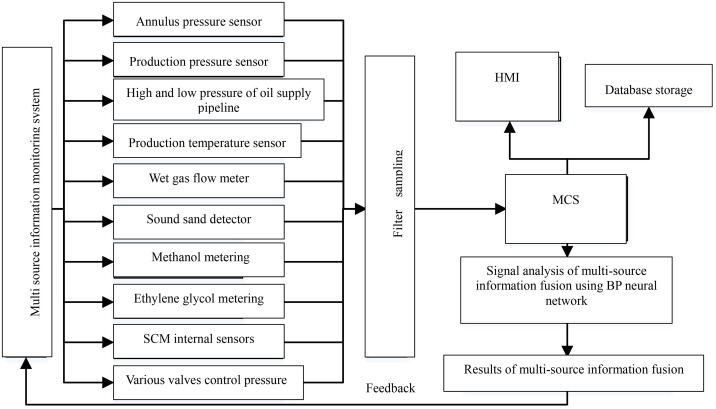
Multi-source information monitoring system process for the Christmas tree.

In the comprehensive evaluation process of multi-source information fusion for the installation, recovery, and in-service production of the Christmas tree, it is necessary to obtain parameter information such as flow rate and pressure through various types of sensors, and then perform correlation, feature processing and state evaluation. Multi parameters fusion algorithms are used to comprehensively process and analyze multiple parameters, accurately identify the operating status of the Christmas tree, in order to timely determine the type of fault and take measures. Based on this, a process model of a Christmas tree monitoring system based on multi-sensor information fusion is established, as shown in Fig 5.

**Fig 6:**
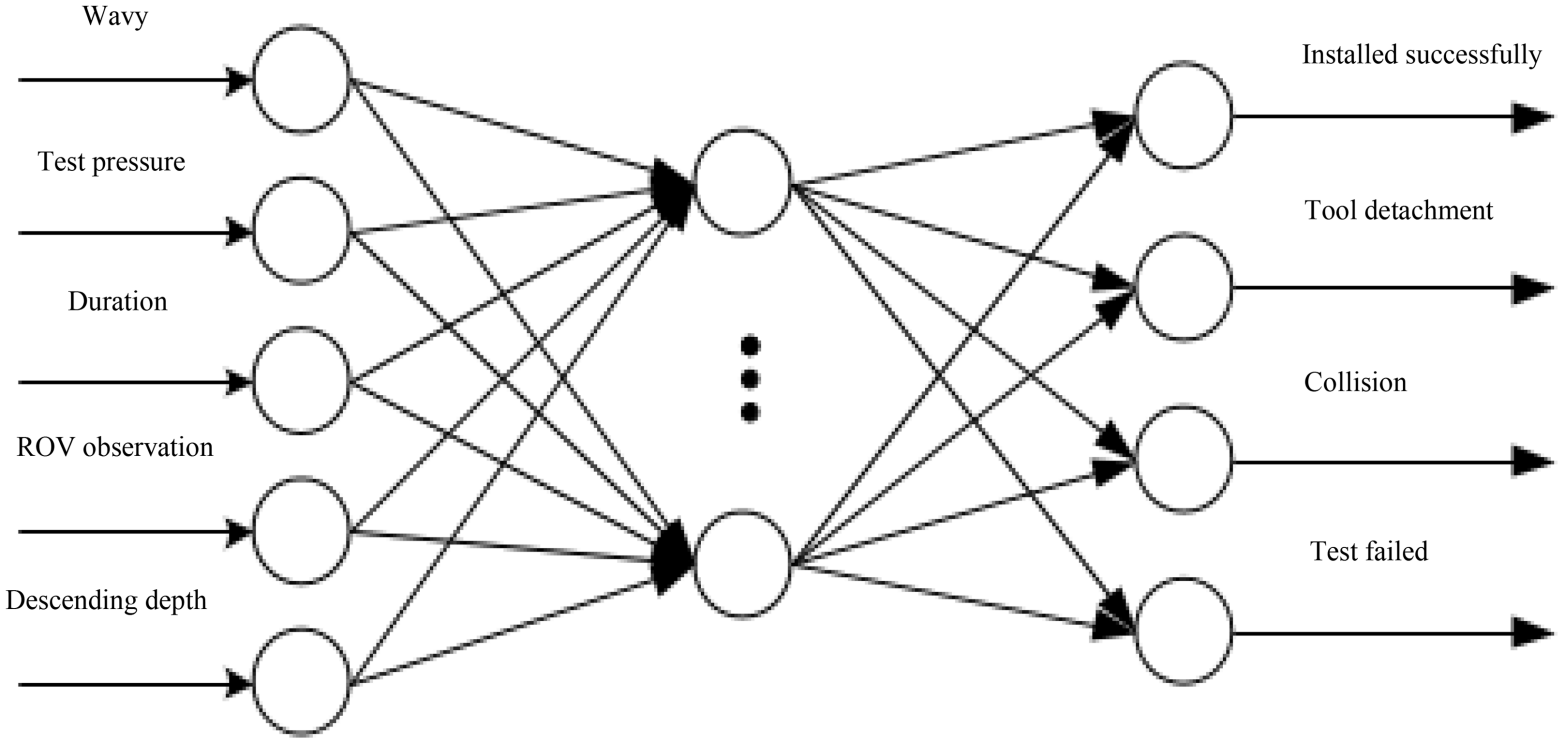
GA-BP neural network fusion model for installation and recovery process of the Christmas tree.

**Fig 7:**
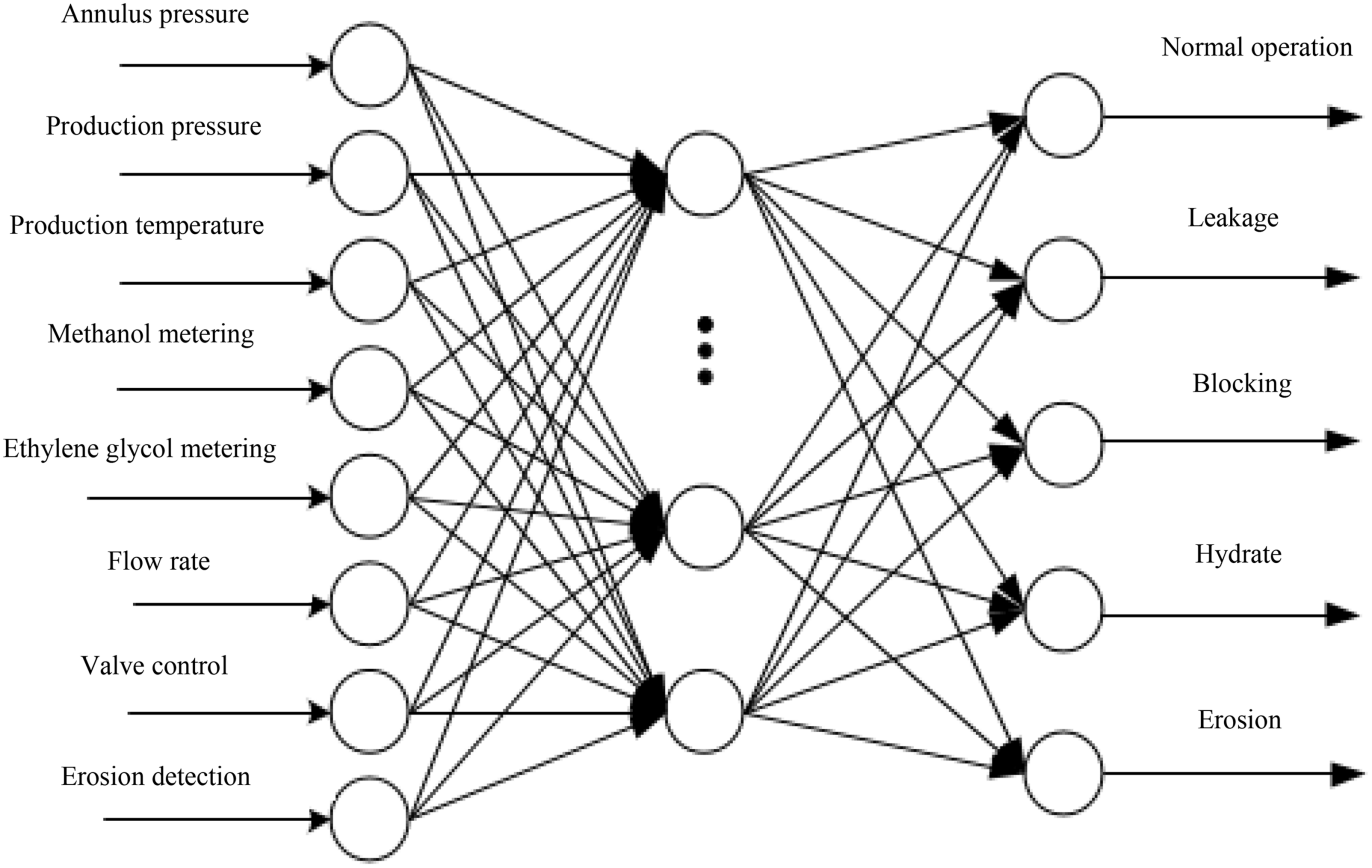
GA-BP neural network fusion model for operation of the Christmas tree.

Based on the different characteristics of sub-sea oil production installation and recovery process and in-service production process, a three-layer network structure is established, and two sub neural networks are constructed, as shown in Figs 6 and 7.

The sub neural network for the installation and recovery process of the Christmas tree is shown in Fig 6. The installation and recovery process involves the use of a drilling platform with dynamic positioning (DP) or a crane on a professional installation vessel to lower the Christmas tree to the predetermined installation position and connect it to the sub-sea wellhead. As the only auxiliary tool, ROV is used to achieve full monitoring and auxiliary positioning, and with the help of its own hydraulic power source, it connects to the fast hydraulic joint Hot Stab to achieve locking by pushing the hydraulic cylinder of the wellhead connector. During this process, the SCM is not connected to the power and communication process and is in an inactive state. Moreover, the installation process is greatly affected by external environmental factors such as waves and currents. Working under suitable wave and current conditions can minimize the impact of environmental factors.

The sub neural network for operation of the Christmas tree is shown in Fig 7. During this process, the pressure, temperature, flow rate, etc. are mainly monitored through sensors on the Christmas tree and SCM, and then the real-time parameters during the production process of the Christmas tree is obtained.

By using the GA-BP neural network fusion model that has been constructed for the installation and recovery process, as well as the operation process of the Christmas tree, and combining it with the GA-BP neural network fusion algorithm, the sample data obtained from the monitoring system is learned and trained. The connection weights between each layer are continuously adjusted until the desired output is obtained ().

**Fig 8:**
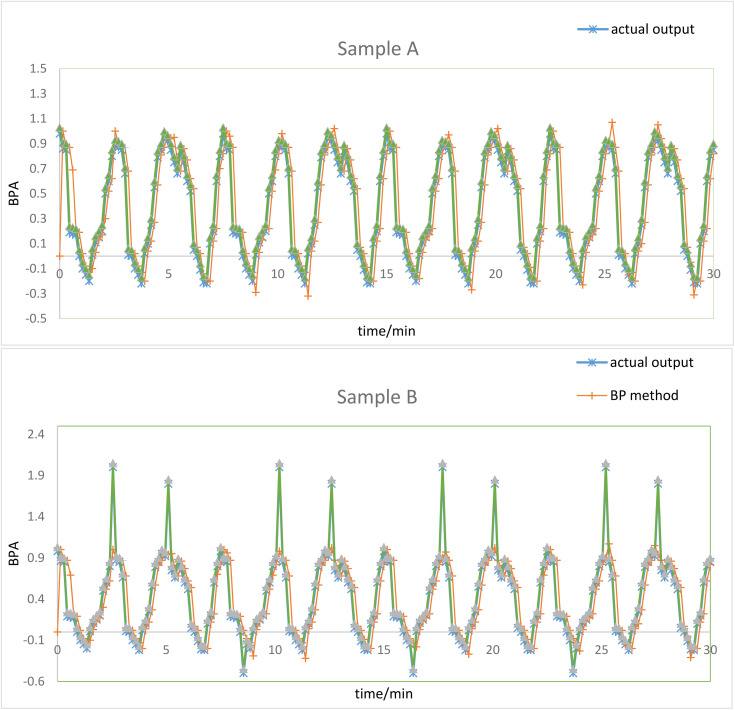
Prediction error curves between different prediction methods and actual values.

**Fig 9:**
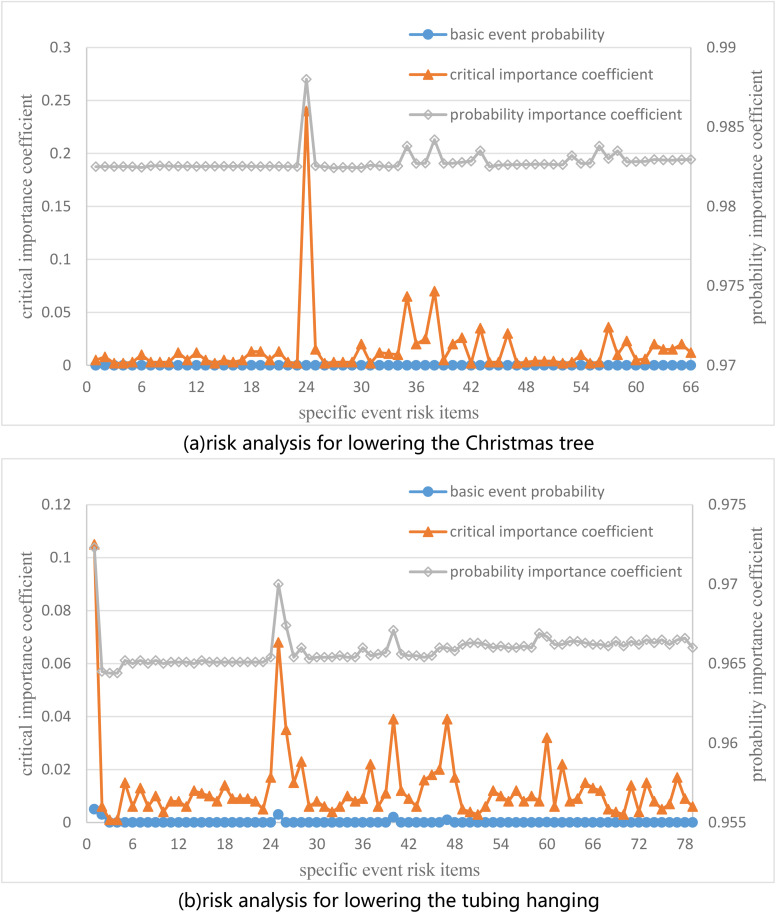
Risk importance coefficient during the installation of Christmas tree and tubing hanger. (a) Risk analysis for lowering the Christmas tree. (b) Risk analysis for lowering the tubing hanging.

**Fig 10:**
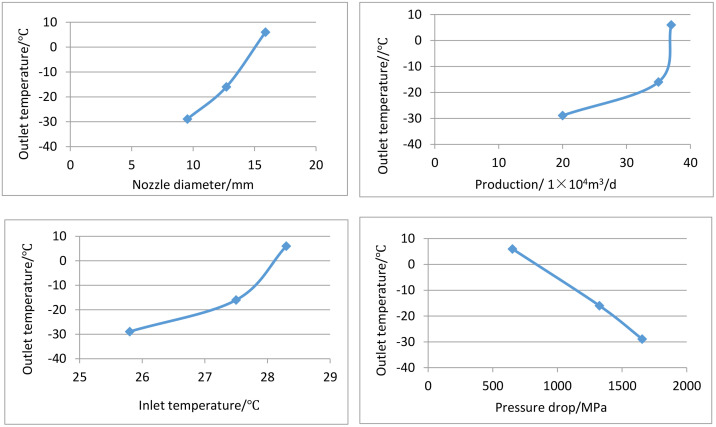
Sensitivity analysis of testing parameters.

**Fig 11:**
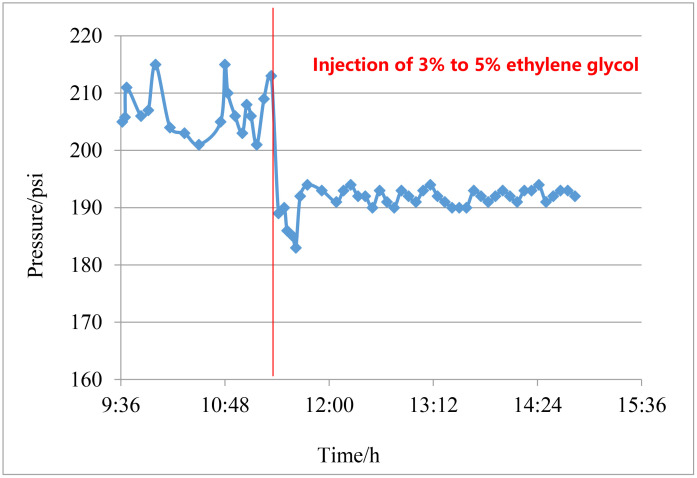
Pressure curve at the choke before and after inhibitor injection.

### 4.2. Simulation analysis

To verify the performance of the GA-BP neural network fusion model, two sets of test training sample data, A and B, were selected for simulation analysis of the model. The training sample collection point is the production pressure 1A on the Christmas tree body, and the sample data is set to normal operation for 30 minutes with a collection interval of 0.15 minutes. Therefore, a total of 202 data samples are used for testing. Similarly, the training sample data for Group B is set to run the Christmas tree for 30 minutes with a collection interval of 0.15 minutes. However, the data sample with a modified production pressure of 1A showed some abnormal pressures during the operation of the Christmas tree, posing a safety risk. The GA-BP neural network structure has 2 nodes in the input layer, 5 nodes in the hidden layer, and 1 node in the output layer, with a total of 15 weights and 6 thresholds. Therefore, the individual encoding length of the genetic algorithm is 21 to control the fitting process.

Combining the above two sets of training data, traditional BP neural network method and GA-BP neural network method are used to predict the accuracy and stability of risk data during the operation of the Christmas tree. The correlation curves between the predicted values and actual values of the two methods are shown in S1 Fig. From S1 Fig, it can be seen that in the two training samples of Group A and Group B, the prediction results of the GA-BP neural network method have smaller errors compared to the traditional BP neural network method and are more accurate in terms of actual output. During the normal operation the relative error between the two prediction methods and the actual output value is relatively small. However, when the Christmas tree experiences abnormal pressure fluctuations, the traditional BP neural network method shows a larger relative error value, with a maximum calculated mean square error of 25%, while the GA-BP neural network method has a maximum error rate of 4.5%.

To verify the accuracy and repeatability of the model, production sample data from 8 other monitoring points on the Christmas tree are continued to be selected for training and testing. The calculation errors of the two models are shown in Table 4. From Table 4, it can be seen that the maximum mean square error occurs at the APT A monitoring point. The reason is that during the production of the well, the wellbore temperature changes rapidly from low to high, causing a rapid increase in the annular trap pressure, resulting in frequent fluctuations in the monitoring data and an increase in the error value. The traditional BP neural network method has an error value of 33.50%, while the GA-BP neural network method has a maximum error rate of 5.85%. However, based on the average training error of samples from 9 monitoring points, it can be seen that the average square error of the model is only 5.10%, which demonstrating excellent stability of the model and verifying the accuracy and superiority of the fusion algorithm.

**Table 4:** Comparison of average errors between BP method and GA-BP method.

No.	Sensor	maximum calculated mean square error/%	
		BP method	GA-BP method
1	CIMV MEG ECM	26.40	4.84
2	CIMV MeOH ECM	22.50	4.30
3	CIMV CI OPT	20.70	4.25
4	WGFM 1	27.50	5.25
5	ASD	29.50	5.30
6	PI1 A	25.00	4.50
7	TI1 A	32.80	6.52
8	APT A	33.50	5.85
9	average	27.24	5.10

### 4.3. Risk analysis

#### 4.3.1. Installation risk analysis.

(1) Classification of installation failure conditions

According to the multi-source information parameters collected during the installation process of the Christmas tree, the failure conditions can be divided into four types: preparation failure, lowering failure, testing failure and recovery failure, as shown in Table 5.

**Table 5:** Classification of failure conditions for Christmas tree installation.

No.	Failure type	Specific event information (66 items)
1	Preparation failure	Mainly includes event information such as lifting deviation, collision with the platform, collision with the moon pool, and failure of wellhead preparation, etc.
2	Lowering failure	Mainly includes event information such as failure of the connection tool, failure of the locking mechanism, failure of unlocking, failure of sensors, and failure of the control mechanism, etc.
3	Testing failure	Mainly includes event information such as high/low pressure line pressure test failure, sealing failure, valve failure, connectivity failure, hydraulic system failure, and testing equipment failure, etc.
4	Recovery failure	Mainly includes event information such as unlocking failure, mechanical control system failure, recovery device failure, rov failure, etc.

(2) Installation risk analysis

Based on the installation failure conditions of the Christmas tree and the fusion analysis of installation process a GA-BP neural network is established. It is found that among the basic failure types, the probability of failure in the preparation for lowering is the highest due to the adverse effects of sea conditions, including preparation work such as lifting deviation, collision with the wellhead, and swinging during lifting. The probability of sealing test failure caused by damage to the VX sealing ring, foreign objects on the sealing surface of the connector and wellhead, etc. is second, while the probability of recovery failure caused by mechanical arm failure, heave compensator failure, and unskilled operation technology is the lowest. Therefore, corresponding measures should be taken during installation, such as paying attention to the influence of sea conditions, lowering speed, cleanliness of the sealing surface, damage to the sealing components, and deviation of the Christmas tree and wellhead, in order to minimize human operational errors and ensure the safe lowering and installation of the Christmas tree system in place. Among them, the 66 specific event risk items probabilities and importance coefficients for lowering the Christmas tree are shown in S2a Fig, and the 79 specific event risk items probabilities and importance coefficients for lowering the tubing hanging are shown in S2b Fig.

#### 4.3.2. Production risk analysis.

(1) Production nodes division

According to the structure and functions of each part of the Christmas tree, this article divides the Christmas tree system during the production process into three nodes: oil and gas production process, chemical reagent injection process, and sub-sea power/communication control process, as shown in Table 6.

**Table 6:** Analysis nodes of the Christmas tree during the production.

No.	Node	Node Description
No.1	Oil and Gas production process	Starting from well bottom, passing through the underwater safety valve, then through the production main valve, and ending at the crossover connector.
No.2	Chemical Reagent Injection Process	Starting from the multifunctional quick connector, passing through the chemical reagent metering valve in the control module, then through the chemical reagent injection control valve, and finally through the one-way valve, and ending at the underwater safety valve.
No.3	Sub-sea Power/Communication Control Process	The control system is a composite electro-hydraulic control system, which requires a power communication system to transmit control signals and monitor information during operation. The power communication process is the process of transmitting control signals downstream and data acquisition signals upstream.

(2) Production risk analysis

According to the simulation experiment prediction data in section 4.2, taking the abnormal fluctuation of training sample data in group B as an example, which caused abnormal pressure during the operation. According to the risk analysis results, when high pressure is detected in the oil and gas production process, if the cause is due to hydrate blockage of the pipeline, the probability of occurrence is C, the severity is 2, and the risk level is M. It is recommended to inject inhibitors timely and carry out hydrate removal operations on schedule, as shown in Table 7. At the same time, the improved HAZOP+risk matrix method is used to evaluate the risks of three nodes in the production process. Through analysis, it is found that no high risk failure modes are found during the production process, but there are 36 moderate risk nodes, mainly related to abnormal opening or closing of various valves during the production process, leakage of valves and pipelines, and blockage of hydrates and sediments. Combined with the operation monitoring parameters of the Christmas tree extracted by multi-source information fusion, the abnormal data points of high risk nodes can be calculated and monitored in reverse, thus comprehensively improving the safety production warning method of the Christmas tree and ensuring the safe operation of sub-sea oil and gas production equipment.

**Table 7:** Risk analysis table for typical nodes during production of the Christmas tree.

Node	Deviation	Possible Causes	Consequences	Safeguards	Probability	Severity	Risk Priority	Recommendations
Operating mode	High Pressure	Production main valve/production wing valve partially closed	Production Decreased; Valve damaged; Channels Corroded	Pressure monitoring device; Valve position indicator (ROV observation); ROV valve handle (ROV can control valve position through it)	B	3	M	Equipped with pressure monitoring devices and high pressure alarms
		Hydrate blockage in pipelines	Production Decreased	Inject chemical reagent (injection position is before the underwater safety valve)	C	2	M	Hydrate removal regularly
		Mud or sand blocking the channel or gathering at valve locations blocking the channel	Production Decreased; Channel pressure increased; Erosion points appear in the channel; Valve damaged	–	B	4	M	Sediment removal regularly; Equipped with sediment monitoring device; Valve design and installation location suitable for sediment producing
	Low temperature	Joule-Thomson effect	Hydrate block channels; Low temperature causes materials deformation and failure	Inject chemical reagent Increase the inlet temperature Increase inlet pressure	B	3	M	Design consideration to reduce the minimum operating temperature
	Changes in oil and gas composition	Increased in moisture content	Hydrate block channels	Inject chemical reagent	B	2	L	–
		Increased in salt content	Channel scaled	–	B	4	M	Regularly sampling and testing the salt content of the produced fluid
	leak	geological disaster	Equipment damaged; Oil and gas leakage	Conduct landslide stability assessment on schedule	B	2	L	–
		fatigue	Environmental pollution	Design considering fatigue failure	B	2	L	–
		Internal erosion of the channel; Corrosion inside the channel; External corrosion of equipment	Equipment damaged; Oil and gas leakage	Design cathodic protection; Regular inspection; Inject anti-corrosion chemical reagents; Develop an erosion prevention plan	B	2	L	–

## 5. Field application

### 5.1. Field risk analysis of hydrate

Well YC-X1 in the western South China Sea has a water depth of 1655m. The well is developed using a sub-sea production system. After the installation of the Christmas tree, well testing is conducted at different production rates with using 9.53 mm, 12.70 mm and 15.88 mm nozzles during the well cleaning and blowout period. By changing the testing parameters, the influence and trend of nozzle diameter, test yield, inlet temperature, and pressure drop at the nozzle on the outlet temperature are analyzed to determine the risk of hydrate formation. The analysis results are shown in S3 Fig.

As shown in S3 Fig, the larger the diameter of the nozzle, the smaller the throttle temperature drop and the higher of the outlet temperature. The larger the output, the smaller the throttling temperature drop, and the higher of the outlet temperature. The higher the inlet temperature, the smaller the throttling temperature drop, and the higher of the outlet temperature. The greater the throttle pressure drop of the oil nozzle, the greater the throttle temperature drop, and the lower of the outlet temperature. Based on the analysis results of the improved HAZAOP method, it can be concluded that measures such as increasing the inlet temperature, reducing the throttling pressure drop, and appropriately increasing the inlet pressure can effectively prevent the precipitation of natural gas hydrates at the throttle point of the oil nozzle.

### 5.2. Field control of hydrate

According to the risk analysis results, hydrate inhibitor injection is carried out in front of the Christmas choke in order to reduce the throttling pressure drop, with using a continuous injection of 3% to 5% ethylene glycol, as shown in S4 Fig, the pressure at the choke fluctuated significantly between 200 and 220 psi before injection of the inhibitor. After injection of the inhibitor, the pressure decreased significantly, and the pressure change curve after the choke tended to flatten over time, indicating the weakening of hydrate blockage phenomenon. The pressure remained stable and the vibration of the surface choke is greatly reduced, which effectively controlled the risk of throttling and spraying at the surface choke during the testing period. Finally, the well testing is safe and smooth, and the test production exceeds 50 × 10^4^m^3^ per day.

## 6. Conclusions

(1) Taking the Christmas tree as an example, the implementation principles of various parameters and status monitoring during the installation and operation of the Christmas tree are analyzed. The risk conditions are described, and the applicability of multi-source information fusion technology in the safety risk analysis for sub-sea oil and gas production equipment is obtained.
(2) Based on artificial intelligence multi-source information fusion state estimation method, the GA is introduced to optimize the initial weights and thresholds of BP neural network. A model of a Christmas tree monitoring system based on multi-sensor information fusion is established. Through simulation experiment analysis, it is found that the average error rate of the GA-BP neural network method constructed in this paper is only 5.10%.
(3) An improved HAZOP method is established by combining the risk matrix analysis method. The evaluation shows that failure probability of Christmas tree due to adverse sea conditions is the highest, with a critical importance coefficient of 0.24, and no high risk failure modes are found during the in-service process, but there are 36 moderate risk points.
(4) The application of hydrate inhibitors in the Christmas tree shows that the multi-source information fusion method established in this paper has accurate data identification ability, and the improved HAZOP method has high accuracy in risk analysis, which can meet the field application requirements. According to the recommended hydrate inhibitor injection measures, the safe operation of the Christmas tree in deep water gas well testing has been effectively ensured.

## Supporting information

S1 Fig Prediction error curves between different prediction methods and GA-BP method. (XLS)

S2 Fig (a) Risk analysis for lowering the Christmas tree. (b) Risk analysis for lowering the tubing hanging.
(ZIP)

S3 Fig Sensitivity analysis of testing parameters. (XLS)

S4 Fig Pressure curve at the choke before and after inhibitor injection. (XLS)
